# Long-term Land Cover Dataset of the Mongolian Plateau Based on Multi-source Data and Rich Sample Annotations

**DOI:** 10.1038/s41597-025-05648-8

**Published:** 2025-08-15

**Authors:** Juanle Wang, Kai Li, Tengfei Han, Yifei Sun, Mengmeng Hong, Yating Shao, Zhichen Sun, Meng Liu, Fengjiao Li, Yuhui Su, Qilin Jia, Yaping Liu, Jiazhuo Liu, Jiawei Jiang, Altansukh Ochir, Davaadorj Davaasuren, Mengqiong Xu, Yamin Sun, Shaopu Huang, Weihao Zou, Feiran Sun

**Affiliations:** 1 State Key Laboratory of Resources and Environmental Information System, Institute of Geographic Sciences and Natural Resources Research, Chinese Academy of Sciences, Beijing, 100101 China; 2 College of Resources and Environment, University of Chinese Academy of Sciences, Beijing, 100049 China; 3 Jiangsu Center for Collaborative Innovation in Geographical Information Resource Development and Application, Nanjing, 210023 China; 4 School of Civil Engineering and Geomatics, Shandong University of Technology, Zibo, Shandong 255049 China; 5 College of Geoscience and Surveying Engineering, China University of Mining & Technology (Beijing), Beijing, 100083 China; 6 School of Marine Technology and Geomatics, Jiangsu Ocean University, Lianyungang, Jiangsu 222005 China; 7 College of Geomatics, Xi’an University of Science and Technology, Xi’an, 710054 China; 8 College of Ecology and Environment, Inner Mongolia University, Hohhot, Inner Mongolia 010022 China; 9 Environmental Engineering Laboratory, Department of Environment and Forest Engineering, School of Engineering and Technology, National University of Mongolia, Ulaanbaatar, 14201 Mongolia; 10 Department of Geography, School of Art and Sciences, National University of Mongolia, Ulaanbaatar, 210646 Mongolia; 11 School of Resources and environment, Institute of Disaster Prevention Science and Technology, Sanhe, 065201 China

**Keywords:** Environmental sciences, Environmental impact

## Abstract

The Mongolian Plateau (MP), with its unique geographical landscape and nomadic cultural features, is vital to regional ecological security and sustainable development in North Asia. Existing global land cover products often lack the classification specificity and temporal continuity required for MP-specific studies, particularly for grassland and bare area subtypes. To address this gap, a new land cover classification was designed for MP, which includes 14 categories: forests, shrubs, meadows, real steppes, dry steppes, desert steppes, wetlands, water, croplands, built-up land, barren land, desert, sand, and ice. Using machine learning and cloud computing, the novel dataset spanning the period of 1990–2020. Random Forest algorithm was employed to integrate training samples with multisource features for landcover classification, and a two-step Random Forest classification strategy to improve detail land cover results in transition regions. This process involved accurately annotating 64,345 sample points within a gridded framework. The resulting dataset achieved an overall accuracy of 83.6%. This land cover product and its approach has potential for application in vast arid and semi-arid areas.

## Background & Summary

The Mongolian Plateau (MP), located in the highlands of Eurasia, forms a relatively independent geographical unit with the Baikal Lake Basin to the north, the Altai Mountains to the west, the edge of the East Asia monsoon region to the east, and the Gobi Desert to the south. The MP is a biodiversity hotspot, ranging from the Siberian taiga to the Asian desert steppes, with diverse geographical landscapes shaped by a long-standing blend of nomadic culture and modern agricultural civilization. The characteristics of the MP include the rapid urbanization of central cities in conflict with the environmental carrying capacity, close coupling of regional economic development with trans-Siberian transportation, a single pillar industry of animal husbandry and mineral resource development, transboundary sandstorms, dzud, and desertification risks. Thus, the MP holds great significance for comprehensive land use and land cover change studies and is a hotspot for research on natural resources, the environment, ecology, disasters, and sustainable development in arid and semiarid areas.

Land-cover data are the most fundamental data resource for research on the land surface dynamics of MP. They provide information on the natural state of the surface and can be used for land degradation assessment^1^, ecological environment monitoring^2,3^, global environmental change^4,5^, and sustainable development evaluation^6^. Numerous studies have focused on the land cover in MP, especially in the field of ecological environments. Using the literature and land cover change data, Kempf (2022) reconstructed historical land cover changes and environmental data for Northern China and Mongolia, revealing that the arid regions in the area experienced severe desertification in the 20th century and that land degradation was further aggravated by overgrazing and resource extraction^7^. Zhang *et al*.^8^ evaluated the sustainability of MP development over the past 30 years using land cover data and related indicators of Sustainable Development Goals (SDGs) and pointed out that the degree of land use increased annually in the MP^8^. Luo *et al*.^9^ analyzed the concentration of microplastics in lake water within different environmental regions of the Inner Mongolia Plateau, including deserts, farmlands, grasslands, forests, and meadows^9^. They found that microplastic distribution was positively correlated with farmland and artificial surface cover indicating human activity-related land cover types may exacerbate microplastic pollution in lakes. All of these studies relied on high-quality land-cover products in the MP.

Numerous land cover products have been developed globally, including the European Space Agency (ESA WorldCover)^10^, GlobalLand30 by the National Geomatics Center of China^11^, Copernicus Global Land Cover (GLC100, GLC200) by the European Commission^12^, Global Land Cover Characterization (GLCC) by the US Geological Survey^13^, and Dynamic World by Google^14^. However, these products often have limitations related to data inconsistencies and limited applicability to the MP region. Classification systems for these global data products have not been sufficiently detailed. Areas with finer category divisions were mostly limited to forest types, such as coniferous forests, broadleaf forests, and mixed forests. Details such as meadows, real steppes, dry steppes, desert steppes, deserts, sand, and barren lands, which are common in arid and semi-arid regions, are difficult to reflect. Additionally, these products do not cover a sufficiently long time span to reflect significant change dynamics. In recent years, climate change and human activity have exacerbated the ecological vulnerability of MP. Targeted land-cover products can better analyze issues such as desertification and land degradation in the MP. Consequently, some researchers have developed regional land-cover products specifically for MP. Wang *et al*. (2019) developed a land-cover mapping method based on object-oriented approaches to obtain land-cover data for Mongolia for 1990 and 2010 and updated the data in 2022 and 2023^15,16^. However, these datasets are limited to Mongolia. In addition, products mainly based on human-computer interaction methods lack automatic updating and scale-up expansion capacity. In the era of artificial intelligence and cloud computing, methods based on sample point annotations are more scalable. For example, the GLanCE (Global Land Cover Estimation) land-cover sample set provides two million land-cover sample points worldwide from 1984 to 2020, covering 13 secondary categories^17^. Remelgado *et al*.^18^ collected over 8,000 cropland data samples from Central Asia between 2015 and 2018 and compiled samples of 40 crop types^18^. Fritz *et al*.^19^ utilized crowdsourcing to obtain global land cover data from the Geo-Wiki platform for 10 land cover types^19^. These sample annotation methods can continuously accumulate multiple types of land-cover data, supporting long-term environmental monitoring and land management applications.

However, owing to issues such as time, region, scale, and classification systems, existing land-cover datasets cannot fully meet the research needs of surface-cover changes specific to the MP. To address this challenge, this study proposes a method that integrates machine learning and cloud computing to develop a comprehensive land-cover dataset for MP. This study establishes a comprehensive sample database of the current MP surface cover. Using the Google Earth Engine (GEE), we construct feature bands, train models. and generate land cover data for the growing seasons from 1990 to 2020 in the MP. This dataset is expected to support a wide range of research applications, including resource investigation and utilization, land degradation and desertification monitoring, natural disaster prevention and control, ecological security assessment, and regional sustainable development in the MP or other arid and semi-arid areas.

## Methods

### Study area

**Fig. 1:**
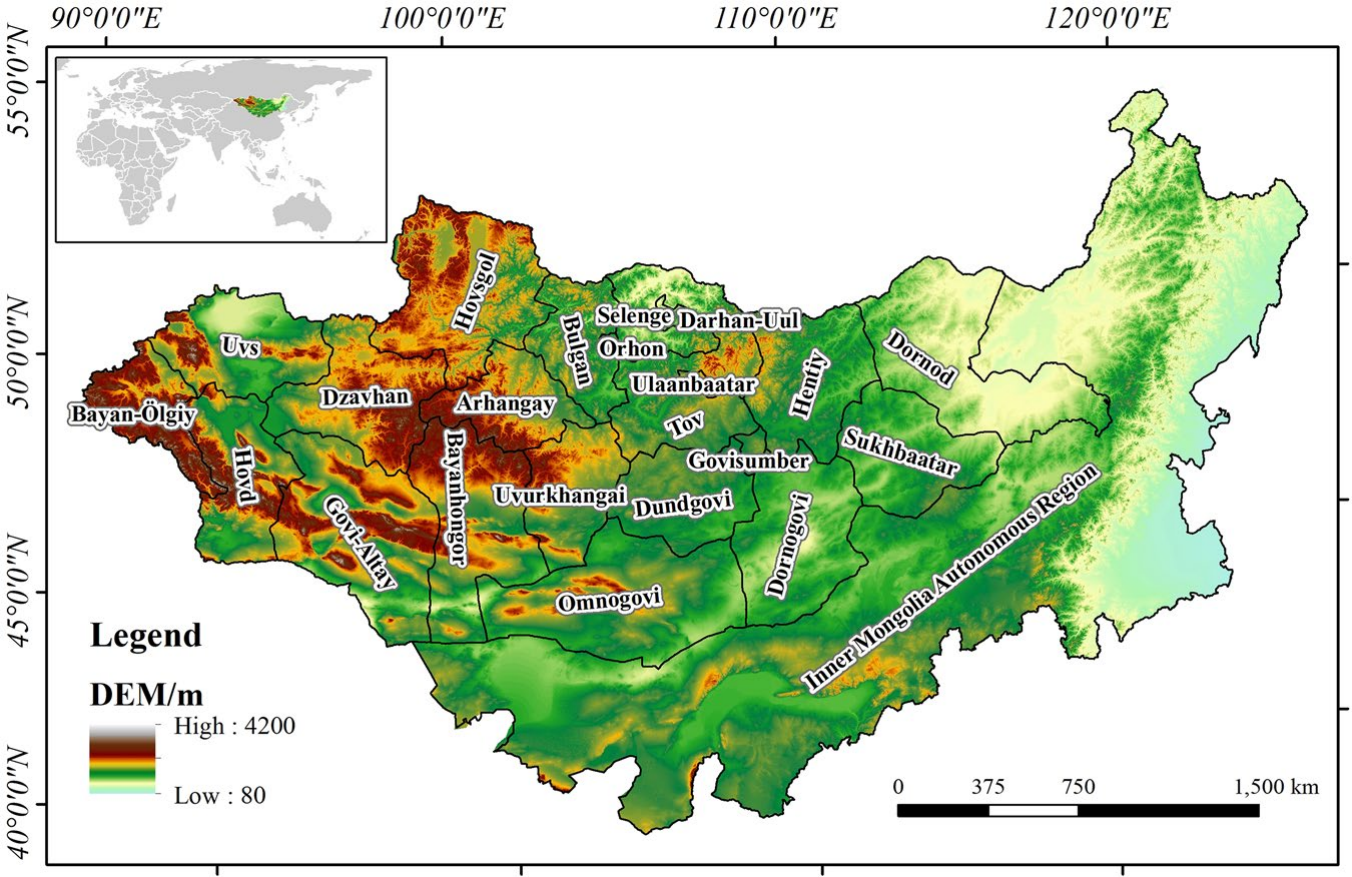
Study area.

The MP encompasses Mongolia and Inner Mongolia Autonomous Region of China. It covers a total area of 2.7 million km^2^, with elevations ranging from approximately 80 to 4,200 m (Fig. 1). The elevation of the MP generally decreases from northwest to southeast. The Altai, Khangai, and Khentii mountains in the northwest of the study area have higher terrain, with an average elevation of up to 3,000 m. The Greater Khingan Range in Inner Mongolia is divided into two parts with significant differences in elevation. The western part generally has an elevation above 1,000 m, whereas the eastern part has an elevation below 500 m. The surface cover types of MP are diverse but primarily consist of grasslands, bare areas, and forests.

### Data source

The data sources are listed in Table 1. Landsat 5 and 8 were used as base maps to classify the respective years. During annotation of the sample points, NASDEM, ESA WorldCover, OpenLandMap Sand Content, and global wetland distribution data points were used to assist in identification. Landsat 5, Landsat 8, NASDEM, and global building volume data were used to build feature bands, train sample points, and produce land-cover data.

**Table 1:** Data Sources.

Data Source	Band Name	Value	Time	URL or Link in GEE
Landsat 5	B1, B2, B3, B4, B5, B7, QA_PIXEL	1–65,455	1984–2012	LANDSAT/LT05/C02/T1_L2
Landsat 8	B2, B3, B4, B5, B6, B7, QA_PIXEL	1–65,455	2013–present	LANDSAT/LC08/C02/T1_L2
NASADEM	elevation	80–4,200	2000	NASA/NASADEM_HGT/001
GHSL: Global building volume 1975–2030	built_volume_total	/	1975–2030	JRC/GHSL/P2023A/GHS_BUILT_V
ESA WorldCover	/	10–100	2021	ESA/WorldCover/v200
OpenLandMap Sand Content	b0	1–100	1950–2018	OpenLandMap/SOL/SOL_SAND-WFRACTION_USDA-3A1A1A_M/v02
Global wetland distribution data points	/	/	/	https://rsis.ramsar.org/

**Fig. 2:**
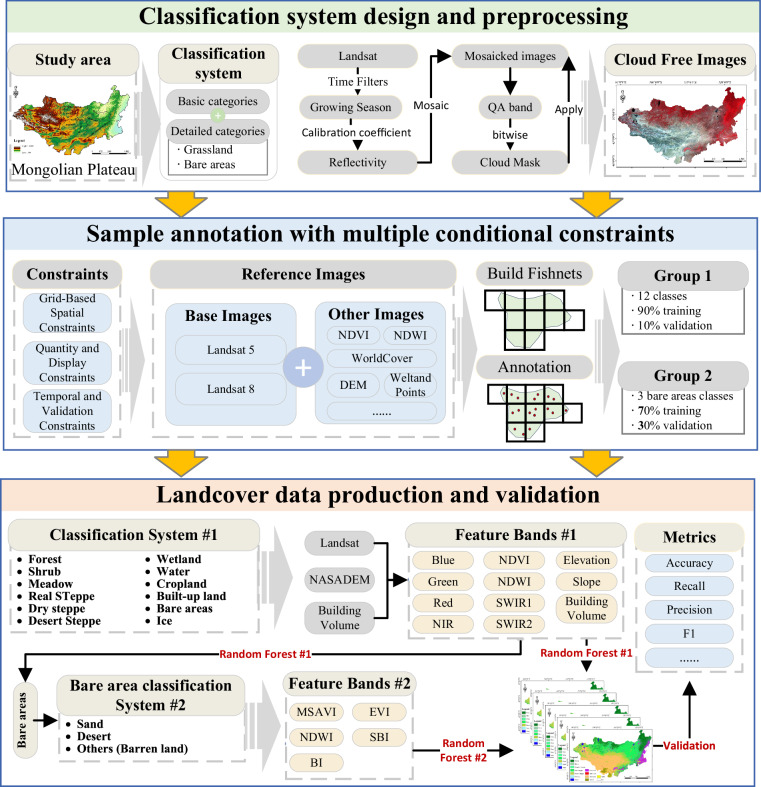
Overall workflow.

This study employed a two-step Random Forest classification approach integrated with machine learning and cloud computing on the GEE to generate a detailed land-cover dataset for the MP from 1990 to 2020 (Fig. 2). The methodology begins with designing the classification system. MP land cover was divided into 14 categories. Landsat 5 and 8 images from the growing season (June 1–August 31) were preprocessed by applying cloud removal, mosaicking, and interpolation to create high-quality annual composites. Subsequently, 64,345 sample points across 14 land-cover categories were manually annotated carefully within a 0.8° × 0.5° gridded framework, ensuring uniform coverage, and validated through dual-annotator consistency checks. Totally 19 partners with the background of geography and remote sensing knowledge joined the cross-checking process, ensuring any sub-regions can be doubled checked. We conducted field verification in MP in recent years, covering whole Inner Mongolia and the eastern, southern, and northern Mongolia. The annotators have a profound understanding of the transition features of land cover throughout the MP. The *in-situ* images and samples from field work helped the annotators to label samples more accurately. The classification process first uses a Random Forest model to categorize the MP into 12 initial classes—including a composite “bare areas” class—based on multi-source features (spectral bands, NDVI, NDWI, elevation, and building volume). In the second step, a refined classification was followed by dividing bare areas into desert, sand, and barren land using additional features. Accuracy validation was conducted using a confusion matrix on the reserved validation samples to assess overall accuracy, precision, recall, F1 score, and kappa coefficient. This approach produces a dataset with seven time points at five-year intervals, tailored for ecological monitoring and sustainable development in arid and semiarid regions.

### Classification system design and preprocessing

**Fig. 3:**
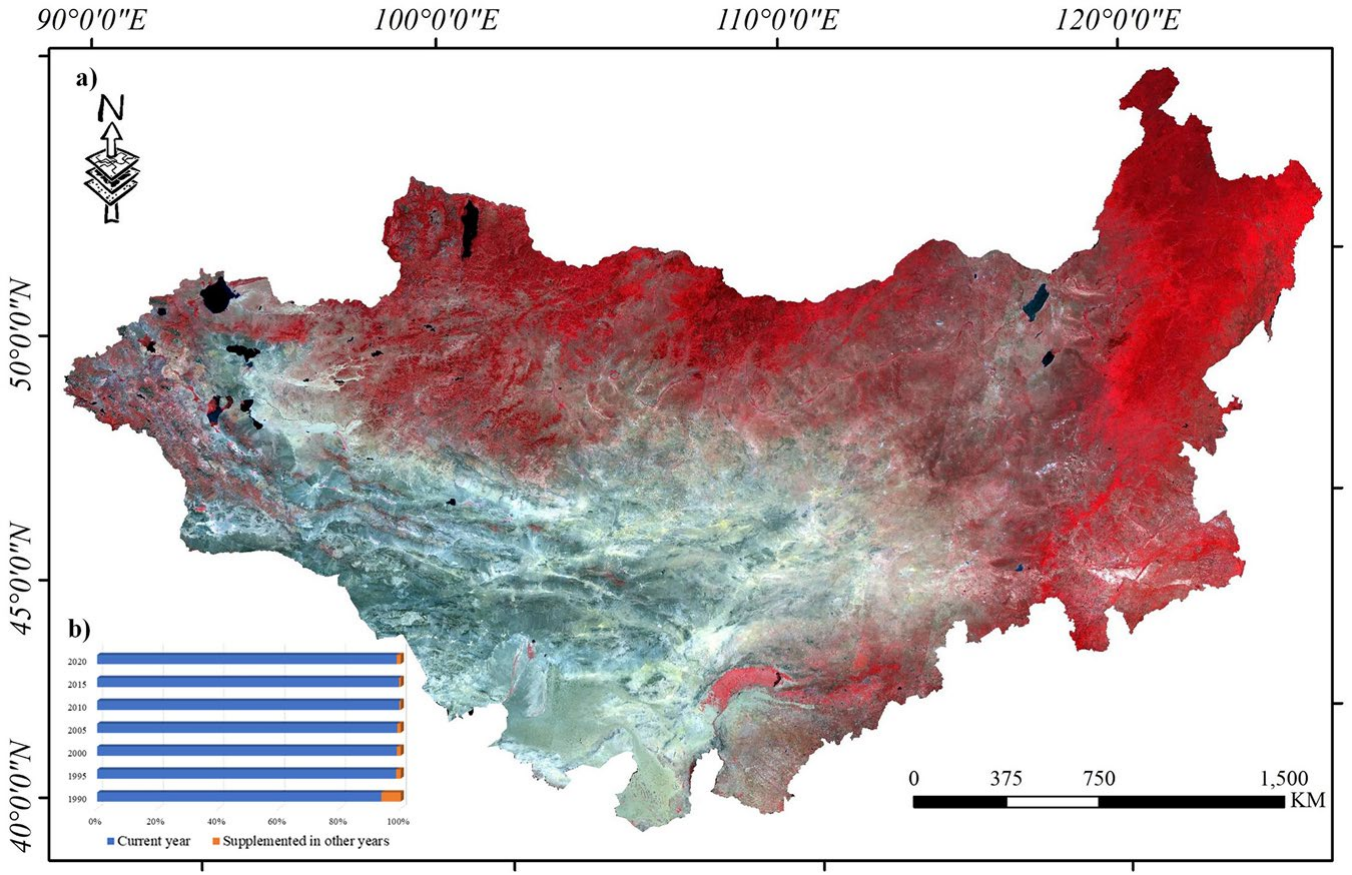
Mosaicked Image in 2020.

This study aims to further improve the land cover system of the MP based on an initial study by expanding the spatial range to the entire MP, enhancing the temporal continuity of data, and supplementing it with more specific land cover types. This study divided MP into 14 categories: forest, shrub, meadow, real steppe, dry steppe, desert steppe, wetland, water, cropland, built-up land, barren land, desert, sand, and ice. The classification system is shown in Table 2, and the reference images for each category are shown in Table S1. Images from 1990 to 2020 were selected at five-year intervals as the base image data. Because Landsat 8 only began collecting data in 2013, data from Landsat 8 were used for 2015 and 2020, whereas data from Landsat 5 were used for 1990, 1995, 2000, 2005, and 2010. For each year, images from the growing season (June 1–August 31) were selected for the composting process. Each band of the Landsat images was assigned a radiometric correction factor to convert DN values into reflectance values. The reflectance images were mosaiced after applying a cloud mask. QA band was used to perform bitwise operations to create masks for clouds and cloud shadows, thereby eliminating areas affected by interference. To address the data gaps caused by cloud cover, we followed a set of strict principles to minimize potential bias. First of all, we used images from the one year before and after the target year for interpolation avoiding long temporal bias. Secondly, to match the target period, only imagery from June to August was used to reduce seasonal bias. Thirdly, mean composite method was employed to reduce the influence of any single year’s anomalies. Figure 3b shows the proportions of interpolation and image area for the current year. The proportion of most interpolation images was less than 2% of the overall area, with only one oldest record in 1990 less than 6.5%. The interpolation areas for other years were less than 2% of the overall area. Given the five-year data span, classification bias from very limited images interpolation can be negligible. Finally, a cloud-free image of the MP was obtained during the growing season. Figure 3a shows the false color display results for the 2020 growing season images.

**Table 2:** Classification system of Mongolian Plateau.

Class		Label	Description
Forest		1	Dense tree canopy with tall trees (canopy height greater than 2 meters), forming a distinct forest ecosystem.
Shrub		2	Discontinuously distributed low-canopy shrublands in Mongolia, typically dominated by sea buckthorn, camel thorn, and Caragana, among others.
Grassland	Meadow	3	Areas dominated by herbaceous plants with relatively uniform vegetation cover, typically growing in moist or fertile soils, characterized by high biodiversity.
	Real steppe	4	Areas dominated by herbaceous plants with vegetation cover ranging between 20% and 50%, rich in plant species diversity.
	Dry steppe	5	Regions with low annual precipitation, where vegetation consists mainly of drought-tolerant herbaceous plants, and the ecosystem is relatively fragile.
	Desert steppe	6	Transitional zones between dry steppe and desert, with sparse vegetation primarily composed of drought-resistant grasses and low-growing plants.
Wetland		7	Areas with long-term or seasonal water accumulation, featuring unique vegetation types such as aquatic plant and marsh plants.
Water		8	Includes all natural or artificial surface water bodies, such as rivers, lakes, ponds, reservoirs, among others.
Cropland		9	Man-made land used for agricultural production, typically showing clear signs of crop cover and tillage.
Built-up land		10	Human-developed land (impervious surfaces), including urban and rural residential areas, industrial zones, transportation infrastructure, etc.
Bare areas	Barren land	11	Bare areas with a soil surface and less plant growth. The surface of barren land is usually composed of mudflats, sandy soil, rocks, or gravel.
	Desert	12	Located in arid and extremely arid regions. Deserts typically cover vast areas with complex aeolian landforms, featuring widespread mobile sand dunes, sparse vegetation, and abundant sand sources.
	Sand	13	Distributed in semi-arid and some semi-humid areas. Sandy areas are smaller in extent, with simpler wind-eroded landforms and sparse vegetation.
Ice		14	Areas with permanent or seasonal snow/ice, including glaciers, permafrost, and snow-covered regions, typically located in high-altitude or high-latitude areas.

### Sample annotation with multiple conditional constraints

Annotation was conducted in two subregions: Mongolia and Inner Mongolia. A label collection team was established to collect land-cover label samples for Mongolia in 1995 and 2020 as well as for Inner Mongolia at five-year intervals from 1990 to 2020. To ensure that the sample points were evenly distributed across the MP, the region was divided into 803 grids of 0.8° × 0.5° (470 and 333 grids for Mongolia and Inner Mongolia, respectively). For each grid, visual interpretation was performed using Landsat remote sensing images from the corresponding year to annotate the land cover type. During data collection, the screen displayed no more than two grid cells at a time and at least 6–8 sample points were marked per grid and distributed relatively evenly. Regional samples for each period were independently collected by two personnel who served as mutual controls. A total of 64,345 samples were collected: 13,378 in 2020, 6,444 in 2015, 8,006 in 2010, 8,126 in 2005, 7,795 in 2000, 13,369 in 1995, and 7,227 in 1990.

**Fig. 4:**
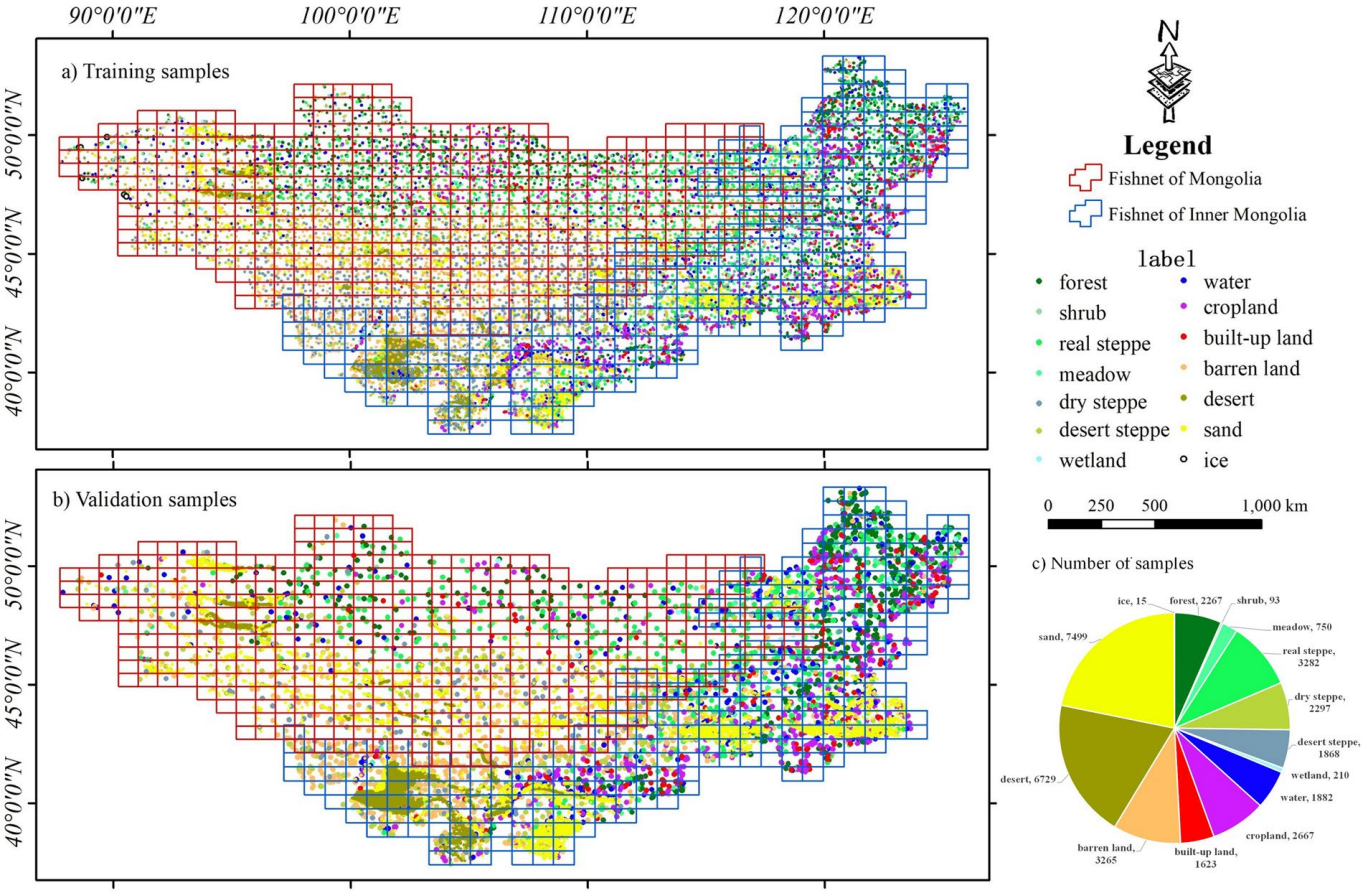
Distribution of labels in each category.

The complexity of land cover types in the MP, particularly the spectral and ecological similarities among bare area subtypes, such as desert, sand, and barren land, poses significant challenges for accurate classification using a single-step approach. A two-step classification strategy was adopted to enhance precision and detail: the first step broadly categorizes land cover into 12 primary classes, including a composite “bare areas” class (Class #1), forest, shrub, meadow, real steppe, dry steppe, desert steppe, wetland, water, cropland, built-up land, bare areas, and ice. Ninety percent of the sample points were used for model training (Fig. 4a), and ten percent were used for product accuracy validation (Fig. 4b). The second step refined bare areas category into distinct subtypes (Class #2). Class #2 represents an independent classification of bare areas subdivided into three distinct categories: desert, sand, and others. The training and validation for the ‘sand’ and ‘desert’ subtypes within bare areas were carried out using a refined 7:3 ratio. This hierarchical method leverages an initial coarse classification to reduce computational complexity and noise, followed by targeted refinement using additional features (MSAVI (Modified Soil-Adjusted Vegetation Index), EVI (Enhanced Vegetation Index), NDWI (Normalized Difference Water Index), SBI (Soil Brightness Index), and BI (Brightness Index)), ensuring the robust differentiation of subtle yet critical land-cover variations prevalent in arid and semi-arid regions. The number of annotations for each category is shown in Fig. 4c, with more sample points for forest, real steppe, desert steppe, cropland, and barren land, and fewer sample points for snow, wetland, and shrub because of their more scattered distribution.

### Landcover data production

#### Construction of the feature Dataset

To account for the different land cover types, the feature datasets included blue, green, red, near-infrared, shortwave infrared 1, shortwave infrared 2, NDVI (Normalized Difference Vegetation Index), NDWI, elevation, slope, and global building volume data for Class #1. Spectral bands, including blue, green, red, near-infrared, shortwave infrared 1, and shortwave infrared 2, are highly effective in distinguishing most land cover types because of their sensitivity to variations in surface reflectance across the electromagnetic spectrum. NDVI can contribute to differentiating land cover types strongly associated with vegetation, such as forests, meadows, real steppes, dry steppes, desert steppes, shrubs, croplands, and wetlands. The inclusion of DEM and slope data can effectively reduce the misclassification of water bodies. Built-up land often has significant spectral differences and some similarities with barren land; therefore, the introduction of global building volume data can aid in the classification of buildings, helping to mitigate the misclassification of built-up lands to some extent. For the refined classification of bare areas (Class #2), the dataset was augmented with specialized indices to capture subtle distinctions within this category. MSAVI was employed to better detect sparse vegetation in arid regions by adjusting for soil background effects that may obscure faint vegetative signals. NDWI was included to accurately delineate water bodies, capitalizing on the strong water absorption in the near-infrared spectrum. The EVI provides additional refinement, offering improved sensitivity for distinguishing vegetation types, especially in regions with high biomass where NDVI might saturate. SBI and BI were also incorporated. SBI targets exposed soil, whereas BI highlights bright surfaces such as sand or rocky terrain, aiding in the separation of barren subtypes.

1 $$NDVI=\frac{NIR-Red}{NIR+Red}$$

2 $${NDWI}=\frac{{Grenn}-{NIR}}{{Green}+{NIR}}$$

3 $${MSAVI}=\frac{2({NIR}-{Red})}{({NIR}+{Red}+1)}$$

4 $${EVI}=2.5\frac{{NIR}-{Red}}{{NIR}+6{Red}-7.5{Blue}+1}$$

5 $${\rm{SBI}}={SWIR}1+{Red}$$

6 $${BI}=\sqrt{{{Red}}^{2}+{{NIR}}^{2}}$$

where NIR, Red, Green, and SWIR1 are the near-infrared, red, green and short wavelength infrared 1 bands, respectively, of the remote sensing images.

#### Model training and prediction

The GEE was used as a platform for data collection and model training. Corresponding to the sample labeling, this process was divided into two parts: (first involved classifying land cover into categories, such as forest, shrub, meadow, real steppe, dry steppe, desert steppe, wetland, water, cropland, built-up land, ice, and bare areas (Class #1). The bare areas were further divided into desert, sand, and barren land (Class #2). First, historical sample point data were integrated and divided into training and validation points in a 9:1 ratio. The training and validation points were packaged and stored on the GEE platform. To address potential cognitive differences among sample collection personnel and improve the issue of overfitting, images and data from the seven periods between 1990 and 2020 need to be standardized. As described in Section 3.3.1, the feature dataset was constructed by extracting the 11 feature pixel values of the target year point-by-point and creating a training point set with a one-to-one correspondence between the feature pixel values and label values. Random Forest classifiers are widely used in land cover classification tasks^20,21^, which can handle high-dimensional, nonlinear classification problems and avoid overfitting problems^22^. In land cover classification tasks, the Random Forest has better performance than other machine learning^23^. Moreover, random forest has demonstrated robustness to image noise, making it particularly suitable for remote sensing classification^24^. After comparison above, the Random Forest algorithm was used to train the training point set in this study. The number of trees of Random Forest is defined by empiric value. Studies have shown a large number of trees (>50–100) produces high accuracy for landcover classification tasks^25^. And when the number of decision trees exceeds 100, the improvement in landcover classification results is not significant^24^. Thus, 100 was choose as the number of trees for this study. These features were combined to construct a predicted image set containing eleven feature bands. A trained Random Forest classifier was applied to the predicted image set to produce land-cover data products.

For the subdivision of bare areas, remote sensing indices such as MSAVI, NDWI, EVI, SBI, DSI, BI, and NDVI were calculated to synthesize the images. By integrating spectral indices and false-color composites and based on a comprehensive analysis of the phenological characteristics of sand, deserts, and other land features, the key features for identifying sand and deserts were determined. Sample points were generated through visual interpretation, and the labelled samples were divided into training and testing sets in a 7:3 ratio. Multi-source remote-sensing data, including NASADEM, ESA World Cover, and Sand Content, were combined with topographic features (elevation, slope, and aspect) and gray-level co-occurrence matrices. Using the Random Forest algorithm, sand and desert information for the MP was extracted, resulting in a detailed distribution map of sand and desert areas across the region.

## Results

**Fig. 5:**
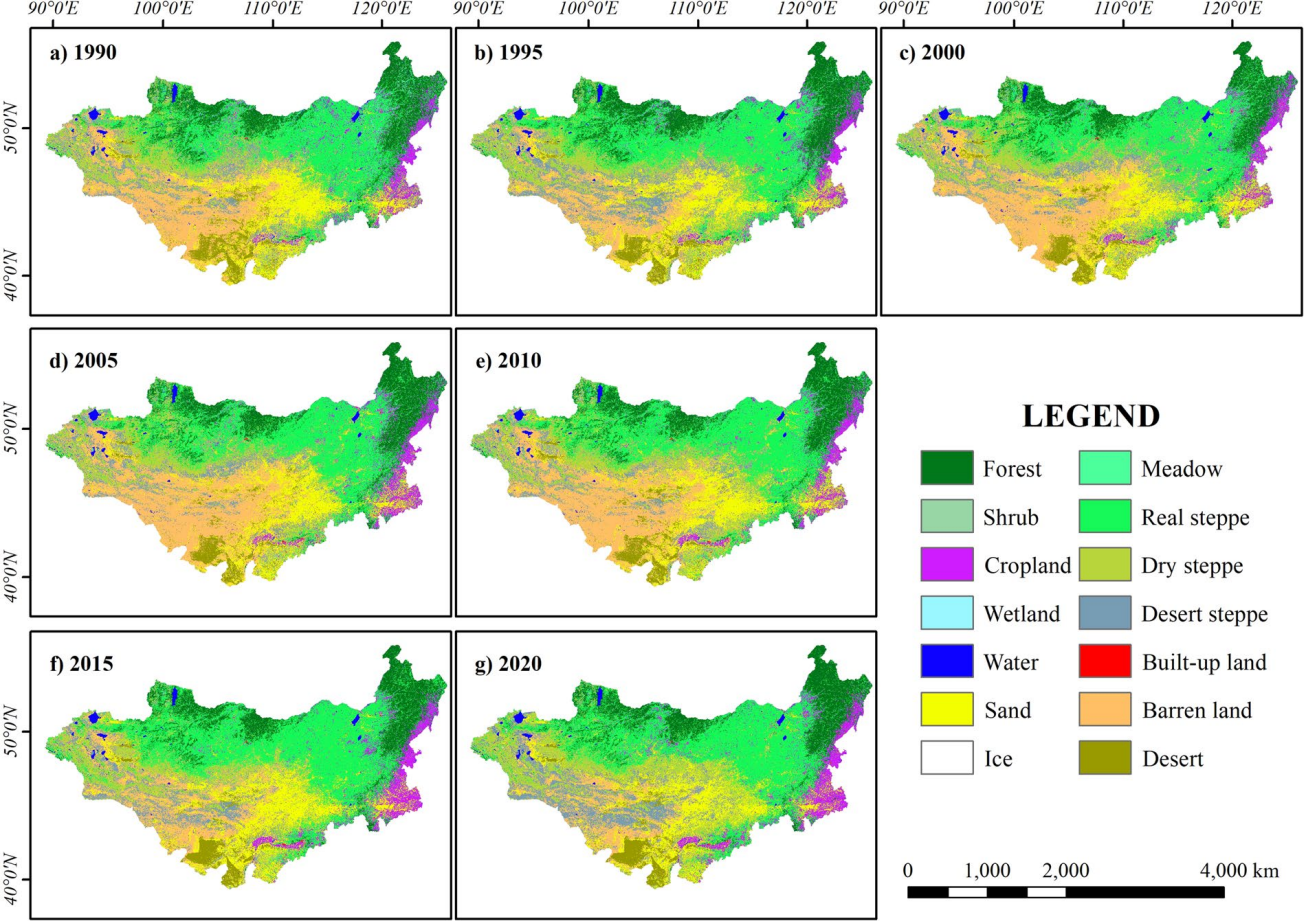
Land cover map of the MP in 1990, 1995, 2000, 2005, 2010, 2015, and 2020.

The MP land cover (Fig. 5) from 1990 to 2020 was dominated by four main types (forests, real steppe, dry steppe, and barren land), with stable distributions over the period. Forests primarily covered the northern regions (45°–50°N, 90°E to 105°E), real steppe dominated the central and eastern areas (40°–45°N, 100°E to 120°E), dry steppe was prevalent in the southern and central regions (40°–45°N, 90°E to 110°E), and barren land was concentrated in the southwestern areas (40°–45°N, 90°E to 100°E). These dominant land covers reflect the geographical and climatic gradients of the region, with minor types such as cropland, shrubs, meadows, desert steppe, and water bodies, which are not significantly dominant. Vegetation cover has increased over the past three decades in general, with forests, meadows, and real steppes in the northern and central regions showing significant recovery, likely due to ecological protection policies or climate change. Desertification was mitigated in the south as barren land, desert, and desert steppe areas decreased, possibly because of environmental management efforts. Agricultural and urban expansion were evident in the southeast, with cropland and built-up land growing consistently, reflecting intensified development. Water body distribution remained stable throughout the study period.

### Trends in land cover change on the MP

**Fig. 6:**
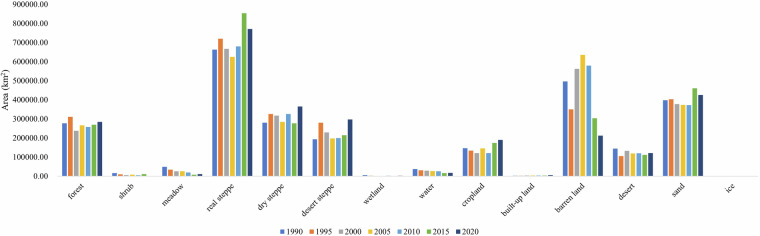
Annual area changes of land cover.

The area statistics for each land cover type were calculated for each year (Fig. 6). The MP was dominated by real steppe, barren land, sand, and forest ecosystems. Real steppe was the most dominant land cover type in the MP, accounting for 26%, followed by barren land, sand, dry steppe, and forest (accounting for 17%, 15%, 11%, and 10%, respectively, from 1990 to 2020), whereas minor categories such as wetlands (0.08%) and ice (0.01%) contributed minimally. These proportions underscore the ecological diversity of the plateau, with grasslands and arid surfaces forming the primary landscapes.Significant shifts occurred over the three-decade period (Table 3), driven by both environmental and anthropogenic factors. The real steppe expanded at a rate of 4,317 km^2^/year, recovering from a low of 624,927 km^2^ (2005) to a peak of 853,368 km^2^ (2015). Concurrently, croplands increased by 1,516 km^2^/year, reaching an average of 147,243 km^2^. In contrast, barren land declined sharply (~ 6,665 km^2^/year), decreasing from 634,974 km^2^ (2005) to 211,699 km^2^ (2020), suggesting land rehabilitation or conversion to vegetated cover. Desert areas also decreased (−496 km^2^/year), which is potentially linked to targeted anti-desertification policies. Forests experienced a gradual decline (−284 km^2^/year) likely due to manmade logging or climate stressors (e.g., wild fire). Meadows and wetlands diminished significantly (−1,263 and −83 km^2^/year, respectively). Interestingly, despite reductions in desert (−496 km^2^/year) and barren land (−6,665 km^2^/year), sandy areas increased by 1,372 km^2^/year, peaking at 459,875 km^2^ in 2015, suggesting complex geomorphological or anthropogenic drivers, including wind-driven sand migration from rehabilitated arid zones, unsustainable land use (e.g., overgrazing), or the encroachment of mobile dunes into previously stable areas.

**Table 3:** Area and change trend of various types of land cover.

Land cover type	Average area (km^2^)	Slope (km^2^/yr)	P value
**forest**	271626.25	−283.59	0.02
**shrub**	7695.78	−354.82	0.02
**meadow**	24490.34	−1262.57	0.01
**real steppe**	711152.90	4317.40	0.02
**dry steppe**	310666.44	1168.37	0.00
**desert steppe**	229876.11	1067.41	0.00
**wetland**	2134.18	−83.01	0.02
**water**	25938.26	−653.32	0.03
**cropland**	147242.96	1516.40	0.00
**built-up land**	2939.38	72.02	0.02
**barren land**	448312.31	−6665.12	0.03
**desert**	121796.71	−495.84	0.00
**sand**	401449.12	1372.36	0.00
**ice**	298.58	−7.48	0.02

More details of sharp shifting of some land cover types were explained as below. From 1990 to 1995, the areas of forest, real steppe, dry steppe, and desert steppe increased significantly. However, this trend reversed between 1995 and 2000. This turning up and down reversal can be mainly attributed to Mongolia’s transition from a socialist to a market-oriented economy in early 1990s, which led to economic slowdown and a decline in industrial output. This economic downturn inadvertently created a window of opportunity for ecological recovery. In contrast, the period from 1995 to 2000 saw intensified human activities in Mongolia, resulting in an obviously reduce in the areas of forest, real steppe, dry steppe, and desert steppe. In this period, the livestock number increased very quickly driven by the economic benefit factor. Recorded by the Mongolia government, the livestock number increased from 28.572 million head in 1995, to 30.227 million head in 2000^26^, and reaching 67.068 million in 2020. The areas with the rapid growth in goat and sheep density are primarily located in Gobi region, the southern part of Mongolia (east of Omnogovi)^27^ (Figure S1d), potentially leading to extensive grazing of dry steppe, resulting in degraded areas. Besides this, a new Minerals Law liberalized the mining sector, leading to rapid expansion—particularly in gold mining, which accounted for 60% of the industry. During this period, the areas of surface coal mining sites increased located around Ordos, Inner Mongolia^28^ (Figure S1e). However, after 2010, the growth trend of coal mining in this region slowed down^28^. The reason for the reduce of forest area in 2000 may be caused by forest fires. It is reported that the forest fire-affected areas reached 10.7 and 12.4 million ha in 1996 and 1997 respectively^29^—more than six times the annual average during the preceding 15-year period (1981–1995). Wildfires predominantly occurred in the forest areas of the northern Selenge River Basin (Figure S1a) and grassland areas of the Hulun Lake Basin^30^ (Figure S1c). A decrease in cropland also occurred near Darkhan in the Selenge River Basin^31^ (Figure S1b). After 2005, barren land decreased substantially, while forest, real steppe, dry steppe, and desert steppe areas generally exhibited an increasing trend. Major contributing factors include the ecological recovering engineering implement in China side, such as the “Three-North” protection forest system construction, and the “Grain for Green” program^32^ in the Inner Mongolia. In the same period, Mongolia’s National Program on Combating Desertification and the National “Green Wall” programs also contribute to the ecological restoration. Moreover, the increase in annual precipitation at this period also played a crucial role in the ecological improvement across the MP.

**Fig. 7:**
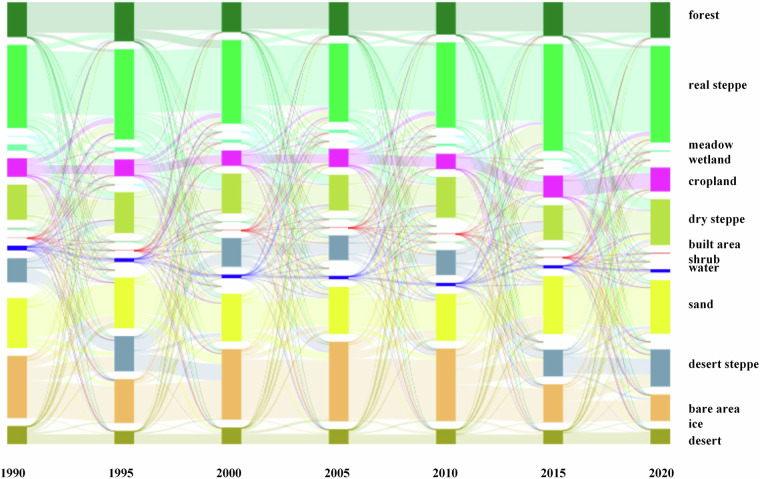
Land cover transitions at 5-year intervals from 1990 to 2020.

From the land cover transitions every five years (Fig. 7), the most significant mutual transitions occurred among the four categories: barren land, desert steppe, real steppe, and dry steppe. Between 1990 and 1995, the largest transition occurred from barren land to desert steppe, covering an area of 112,152 km^2^. Two main trends of land cover change can be observed in the MP: land degradation from 1995 to 2005, followed by land recovery from 2005 to 2020. Between 1995 and 2005, land cover changes primarily reflected in the largest transitions from desert steppe to barren land (1995–2000) and from real steppe to dry steppe (2000–2005). The main factors are decreased precipitation and intensive human activities. Compared to the 1980s, precipitation during this period dropped by as much as 80%, with the most significant decline occurring in central Mongolia—an average annual decrease of approximately 0.4 mm^33^. Economic development is another driving forces from the changes of the GDP data. The GDP growth rate is high in this period, reaching to the highest 10.63% in 2004, and the mineral resource development contributing over 20% to this growth. After 2005, the land cover changes primarily reflected in the largest transitions from dry steppe to real steppe (2005–2015) and from barren land to desert steppe (2015–2020). Summer precipitation increased these years: between 2012 and 2021, the average summer precipitation was approximately 180 mm, significantly higher than the 140 mm recorded during 1999–2011^34^. Ecological restoration policies implemented by China and Mongolia have played a vital role in promoting recovery. It is reported the forest cover increased from 7.73% to 22.1% in Inner Mongolia supported by the forest protection project. Inner Mongolia introduced a series of ecological compensation policies such as grazing prohibition, pasture resting, and rotational grazing. These measures contributed to stabilizing livestock numbers after 2010, thereby reducing pressure on grassland ecosystems^35^. Research indicates that from 2010 to 2015, restored land area across the MP accounted for 26.8% of the total area—1.5 times the area that experienced degradation^35^.

## Data Records

The MP_LULC land cover product, sample set, and corresponding documentation generated in this study are accessible via the Science Data Bank at 10.57760/sciencedb.07381 or https://www.scidb.cn/en/s/3AfYjm^36^. The product is stored in GeoTIFF format and includes “. ovr” pyramid files necessary for opening in ArcGIS,” and dbf: statistical files for each category, among other relevant files. The dataset was packaged as a ZIP file. In the raster data, labels 1–14 correspond to forests, shrubs, meadows, real steppes, dry steppes, desert steppes, wetlands, water, croplands, built-up land, barren land, deserts, sand, and ice, respectively. The samples set were categorized into Class #1 and Class #2, stored in separate files, with each class containing columns for year and land cover label.

To facilitate user access and computation, this dataset is also available online via the Google Earth Engine (projects/ee-carylee/assets/MP_ dataset/Landcover1990_2020). The data were stored in ImageCollection format, containing images for seven periods. Users can filter each year to obtain a dataset for that year.

## Technical Validation

The remaining validation points were used to extract land cover data values for the corresponding years. Accuracy was validated using a confusion matrix. The evaluation metrics for accuracy validation included Overall Accuracy, Precision, Recall, F1 score, and kappa coefficient. These metrics range from 0 to 1, with values closer to 1 indicating a better classification performance. Precision, Recall, and F1 scores were used to evaluate the classification performance of each land-cover type, whereas mPrecision, mRecall, and wF1 (weighted F1) were used to assess the overall classification performance.

7 $$Accuracy=\frac{T}{T+F}$$

8 $$Recal{l}_{i}=\frac{T{P}_{i}}{T{P}_{i}+\sum F{N}_{i}}$$

9 $$Precisio{n}_{i}=\frac{T{P}_{i}}{T{P}_{i}+\sum F{P}_{i}}$$

10 $$F{1}_{i}=\frac{2\cdot {Precisio}{{n}}_{i}\cdot Recal{l}_{i}}{{Precisio}{{n}}_{i}+Recal{l}_{i}}$$

11 $$mRecall=\frac{1}{n}\mathop{\sum }\limits_{i=1}^{n}Recal{l}_{i}$$

12 $$mPrecision=\frac{1}{n}\mathop{\sum }\limits_{i=1}^{n}Precisio{n}_{i}$$

13 $$wF1=\mathop{\sum }\limits_{i=1}^{n}{w}_{i}\cdot F{1}_{i}$$

where *T* is the number of pixels where the labels and predictions are consistent, *F* is the number of pixels where the labels and predictions are inconsistent, *TP*_*i*_ is the number of pixels correctly predicted for category *i*, *n* is the number of categories, *TN*_*i*_ is the number of pixels correctly predicted for all categories other than category *i*, *FN*_*i*_ is the number of pixels incorrectly predicted for category *i*, and *FP*_*i*_ is the number of pixels that belong to category *i* but are predicted for other categories. The normalized weight *w*_*i*_ for each category, derived from their respective class frequencies, is used to compute a weighted aggregate of the corresponding F1 scores. This metric ensures that each category’s influence on the final evaluation reflects its prevalence in the dataset, thereby reducing potential biases in the confusion matrix caused by class imbalance.

Table 4 presents the confusion matrix for all validation samples (except for the secondary classification of bare areas) across the seven periods in the MP dataset, with a total of 5,086 validation samples. Table 5 presents the overall statistical accuracy for each category. The overall validation accuracy was 83.6% with a kappa coefficient of 0.807. The average precision, recall, and wF1 scores are 84.5, 79.6, and 83.5%, respectively. Overall, the accuracy of the land cover data was relatively high. The precision metrics for ice, built-up land, water, and forests exceeded 90%. The Recall metrics for ice, bare areas, forests, and water exceeded 90%. The combined F1 scores for precision and recall indicated that the classification performance for shrubs and sand was poorer than that for other land cover types, with scores below 70%. The F1 scores for the forests, real steppes, water, croplands, bare areas, and ice exceeded 80%. Looking at different years, the overall accuracies for 1990, 1995, 2000, 2005, 2010, 2015, and 2020 were 80.9%, 80.6%, 78.6%, 81.3%, 84.6%, 89.7%, and 91.2%, respectively. The Kappa coefficients were 0.77, 0.77, 0.74, 0.78, 0.81, 0.88, and 0.89. The highest classification accuracies were observed for 2015 and 2020. Within the bare areas, sand and desert categories were added and independently validated (Table 6), with 16,663 validation samples selected. Overall, the validation accuracy for sand and desert was 74.8%, with the desert category showing a slightly higher accuracy than the sand category. In terms of years, the classification accuracies for sand and desert from 1990 to 2020 were 78.6%, 76.7%, 76.7%, 70.6%, 75.3%, 76.0%, and 70.2%, respectively.

**Table 4:** Confusion matrix.

	Forest	Shrub	Meadow	Real steppe	Dry steppe	Desert steppe	Wetland	Water	Cropland	Built-up land	Bare areas	Ice
**Forest**	557		3	34	1		1	2	10			
**shrub**		19		1				1	1		6	
**Meadow**	1		119	23	2	2	2	2	11		7	
**Real steppe**	13		10	686	8	4	1	2	23		22	
**Dry steppe**		3	3	22	362	40		1	8		150	
**Desert steppe**	2			4	8	153		3			26	
**Wetland**	2		2	3			28	2	1			
**Water**	2	1	2	1	2	2		413	8		25	
**Cropland**	3	6	11	41	13	2	1		580		33	
**Built-up Land**	1		2	29	8	5		2	20	155	64	
**Bare areas**	5			5	10	38		5	5		1180	
**Ice**												9

**Table 5:** Accuracy indicators.

	Forest	Shrub	Meadow	Real steppe	Dry steppe	Desert steppe	Wetland	Water	Cropland	Built-up land	Bare areas	Ice
**Precision**	0.951	0.655	0.783	0.808	0.874	0.622	0.848	0.954	0.870	1.000	0.780	1
**Recall**	0.916	0.679	0.704	0.892	0.615	0.781	0.737	0.906	0.841	0.542	0.946	1
**F1 score**	0.933	0.667	0.741	0.848	0.722	0.692	0.789	0.929	0.855	0.703	0.855	1
**Overall Accuracy**		83.6%	**Kappa**		0.807	**mPrecision**		84.5%	**mRecall**	79.6%	**wF1**	83.5%

**Table 6:** Confusion matrix and accuracy indicators of bare areas.

	Sand	Desert	Others
Sand	3291	868	1095
Desert	834	3192	710
Others	616	68	5989
Precision	0.69	0.77	0.77
Recall	0.63	0.67	0.90
F1	0.66	0.72	0.83
Accuracy	74.8%		

Besides the accuracy evaluation above, this land cover results were validated using a third-party dataset for independent assessment. The GLanCE dataset from 1984 to 2020 was used for validation because of their well temporal matching. The Level 1 classification system of GLanCE was used for accuracy assessment between our dataset and GLanCE, with the following category mapping scheme: water corresponds to water; ice corresponds to ice/snow; built-up land corresponds to developed; barren land, desert, and sand are merged and mapped to barren/sparsely vegetated; forest corresponds to tree; shrub corresponds to shrub; and meadow, real steppe, dry steppe, desert steppe, wetland, and cropland are combined and mapped to herbaceous. The validation using the GLanCE dataset (3,758 validation samples) yielded an overall accuracy of 85.2%, wF1 of 85.2%, and a Kappa coefficient of 0.766 (Table S2). The overall accuracy retrieved from the GlanCE training dataset aligns closely with our validation results, demonstrating high precision and reliability of this dataset again.

**Fig. 8:**
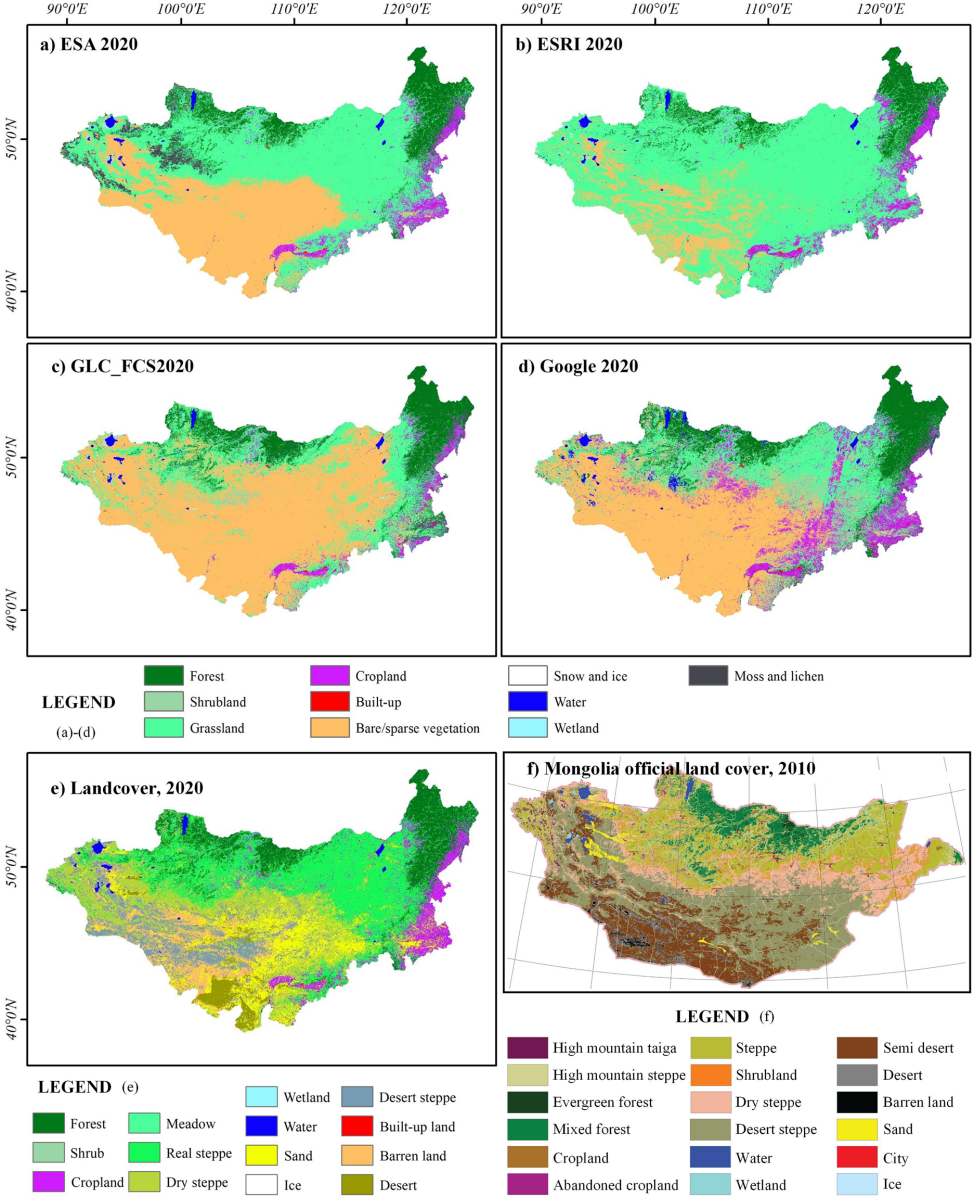
Land cover product in MP: (**a**) ESA WorldCover; (**b**) ESRI landcover; (**c**) GLC_FCS; (**d**) Google Dynamic World; (**e**) Land cover product of this study; (**f**) Land cover map of Mongolia, translate from eic.mn.

Figure 8a–d shows the surface cover of the MP based on global land cover data products (GLC_FCS displays the classification at level 0). In terms of classification systems, the categories were largely consistent, with eight categories present in all four products: Forest, Cropland, Built-up, Grassland, Bare/sparse vegetation, snow and ice, water, and wetlands. Shrubland is included in the ESA, GLC_FCS, and Google Data products. The ESA also includes an additional moss and lichen category that is not found in other products. Although all four data products exhibited a south-to-north transition from bare land to grassland to forest in terms of hierarchical relationships, the differences between the products were significant, particularly in the distribution of grassland. Figure 8f shows the official land cover distribution map of Mongolia. This dataset includes 18 categories characterized by a detailed classification of grassland and bare areas. Grasslands were subdivided into high mountain steppe, steppe, dry steppe, and desert steppe, whereas bare areas were categorized into semi-desert, Desert, Barren land, and sand. However, this dataset is only available for 2000 and 2010, and there are differences in the classification systems between these two years (www.eic.mn). Compared to the official land-cover map of Mongolia, the ESA, GLC_FCS, and Google data products tended to overestimate bare areas while underrepresenting large areas of grassland, with GLC_FCS showing the most severe errors. Additionally, Google Data products misclassified a significant amount of croplands. From the perspective of classification systems, none of these four data products provide detailed secondary subdivisions for grassland and bare areas (GLC_FCS offers secondary subdivisions for bare areas, but its classification system differs from Mongolia’s official system and does not include sand or desert categories). The land cover product we created (Fig. 8e) maintains consistency with global data products in terms of the classification system while also providing a more refined representation of the surface cover in bare areas.

This dataset was a land-cover data product for the MP spanning 1990–2020. It included seven periods (1990, 1995, 2000, 2005, 2010, 2015, and 2020) to describe the changes in the surface cover during the MP growing season. The values ranged from 1 to 14, representing forests, shrubs, meadows, real steppes, dry steppes, desert steppes, wetlands, water, croplands, built-up land, barren land, deserts, sand, and ice. Additionally, the dataset provided label samples for MP for the years 1990, 1995, 2000, 2005, 2010, 2015, and 2020. This dataset can be used for research on land cover changes, related resources, and environmental issues in MP. Considering the local characteristics of MP, a classification system suitable for this region was developed. Secondary classifications, such as meadows, real steppes, and desert steppes, were derived based on grassland classification. In terms of temporal consistency, this dataset has much more long term records and has sustainable potential updating compared with other similar datasets. This can better monitor continuous changes in grass yield, regulate animal husbandry development, and evaluate the ecological service functions in the MP.

For limitations and future study, there still has challenges for the land cover data quality uncertainty analysis and temporal dynamics detection. Firstly, due to the interference of clouds in Landsat data, even if interpolation of previous and subsequent years is used, there may still be unclassified areas covered by clouds and mist for a long time. The differences between Landsat 5 and Landsat 8 sensors may also affect the classification results to some extent. Secondly, for long-term change analysis, the dataset has a long-time record with time interval of 5 years and is suitable for updating and analyzing long-term land cover trends, but may not capture short-term disturbances such as land cover changes caused by extreme weather events or human activities in yearly scale. In the future, multi-source data fusion will be introduced to enhance the reliability of data sources and further optimize the reliability of sample data. Moreover, with the addition of field validation samples, we will further refine the classification system for the MP, including the identification of categories such as the Gobi and abandoned farmland. As the balance of long-term dataset updating and short-term change detection, besides the continually update of this dataset in the future, the CCDC (Continuous Change Detection and Classification) method or Landtrendr method are optional for yearly dataset updating, or annual land surface disturbances and special classification changes detection for further studies.

## Supplementary information

Supplementary materials

## Data Availability

This product was developed using the JavaScript language in GEE, and the code has been uploaded to GitHub (https://github.com/LULC173/MON_LULC). The link for annotating surface features using the fishnet file is “MON_LULC/annotation.js,” the code for classification using Random Forest is “MON_LULC/RF_classification.js,” and the code for visualizing the results is “MON_LULC/read_LULC.js.”
