# Supplementary material for: Long-term Land Cover Dataset of the Mongolian Plateau Based on Multi-source Data and Rich Sample Annotations
Source: Sci Data. 2025 Aug 15;12:1434. doi: 10.1038/s41597-025-05648-8 (PMC12356913; doi:10.1038/s41597-025-05648-8)
Supplement: Supplementary file 1 — Supplementary materials [file 41597_2025_5648_MOESM1_ESM.docx]

Table S1. Confusion matrix from GLanCE training dataset

| Class | | Label | Reference image | Class | | Label | Reference image |
| --- | --- | --- | --- | --- | --- | --- | --- |
| Forest | | 1 | 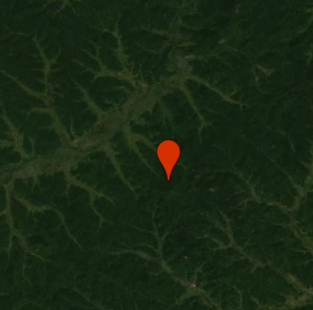 | Water | | 8 | 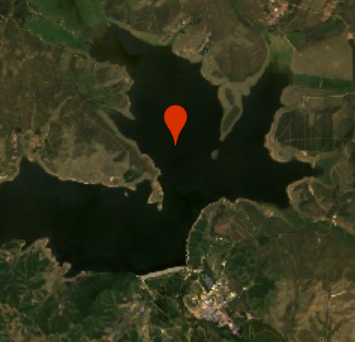 |
| Shrub | | 2 | 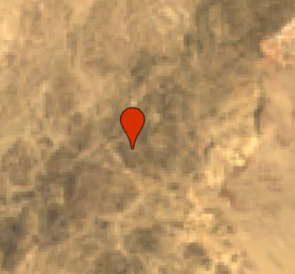 | Cropland | | 9 | 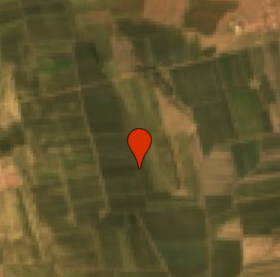 |
| Grassland | Meadow | 3 | 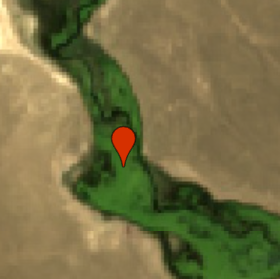 | Built-up land | | 10 | 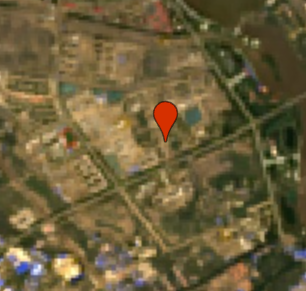 |
|  | Real steppe | 4 | 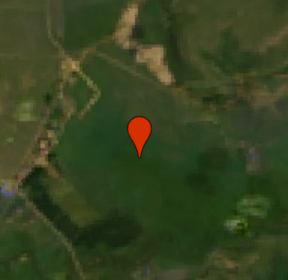 | Bare areas | Barren land | 11 | 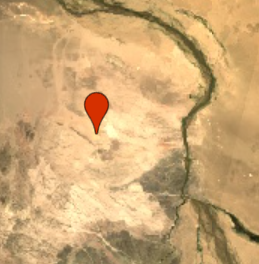 |
|  | Dry steppe | 5 | 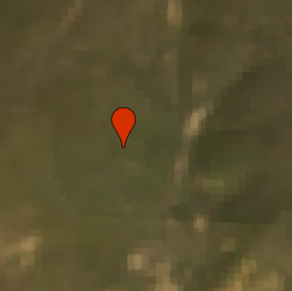 |  | Desert | 12 | 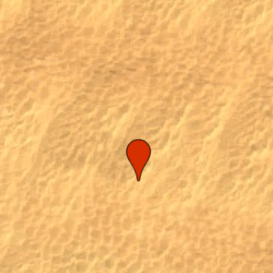 |
|  | Desert steppe | 6 | 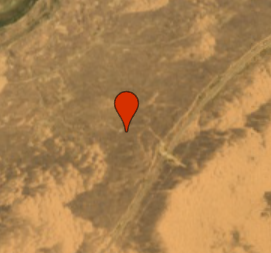 |  | Sand | 13 | 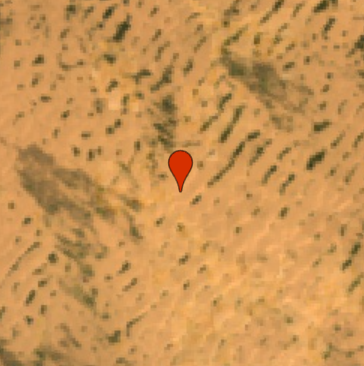 |
| Wetland | | 7 | 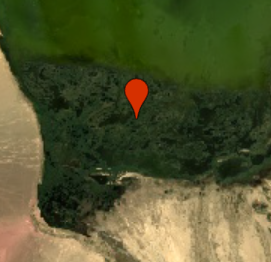 | Ice | | 14 | 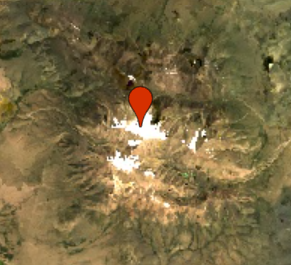 |

Table S2. Confusion matrix from GLanCE training dataset

|  | | **Land cover product of this study** | | | | | | |
| --- | --- | --- | --- | --- | --- | --- | --- | --- |
|  |  | **Water** | **Ice/snow** | **Developed** | **Barren/sparsely vegetated** | **Trees** | **Shrub** | **Herbaceous** |
| **GLANCE samples** | **Water** | 32 |  |  | 1 |  |  | 1 |
|  | **Ice/snow** |  |  |  |  |  |  |  |
|  | **Developed** | 1 |  | 63 | 31 | 1 |  | 17 |
|  | **Barren/sparsely vegetated** | 11 |  |  | 1103 |  |  | 162 |
|  | **Trees** |  |  |  |  | 477 |  | 30 |
|  | **Shrub** |  |  |  | 5 |  |  |  |
|  | **Herbaceous** | 9 | 2 | 1 | 234 | 41 | 10 | 1526 |
| Overall Accuracy: 85.2% | | Kappa: 0.766 | | mPrecision: 83.8% | | mRecall: 82.8% | | wF1: 85.2% |


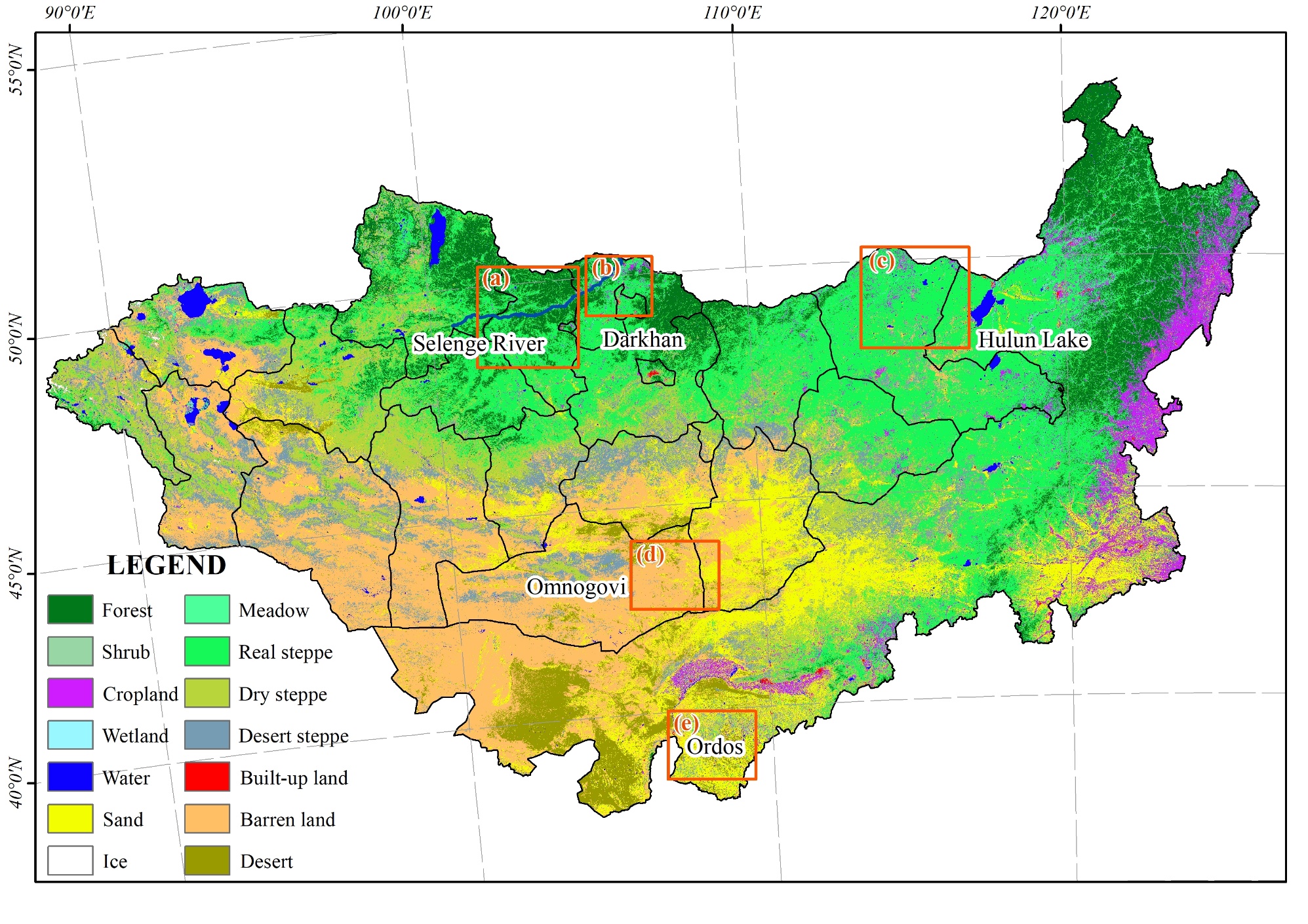


Figure S1. Representative areas of change in fire occurrence, cropland, and livestock populations, and mining on the Mongolian Plateau. (The basemap is land cover map in 2000; (a) & (c) Fire-affected area in 1996 and 1997; (b) Areas of cropland decrease; (d) Areas of increased goat and sheep density; (e) Areas of increased surface coal mining)
